# On the rocky road to efficient behavior management: Can emotional competencies signal the better way?

**DOI:** 10.3389/fpsyg.2022.1049617

**Published:** 2022-12-21

**Authors:** Philippe Gay, Philippe A. Genoud, Gabriel Kappeler, Marilena Cuozzo, Jean-Marc Gomez, Malika S. Bapst, Marina Fiori

**Affiliations:** ^1^Haute Ecole Pédagogique Vaud, UER EN, Lausanne, Switzerland; ^2^Université de Fribourg, Fribourg, Switzerland; ^3^Haute Ecole Pédagogique du Valais, St-Maurice, Switzerland; ^4^Haute Ecole Fédérale en Formation Professionnelle, Lausanne, Switzerland

**Keywords:** emotional competencies, classroom management, behavior management, elementary school, identification of emotions, understanding of emotions

## Abstract

Self-efficacy beliefs in behavior management (SEBiBM) is a key issue for teachers, while emotional competence is a major contributor to professional success and sustainability in this profession. The investigation of the multifaceted nature of these two constructs may be important in order to take a step toward understanding which emotional competence could foster specific aspects of SEBiBM. To explore this issue, elementary school teachers (*N* = 121, 1st-4th grades) answered the Profile of Emotional Competence, which comprises 12 scores of emotional competencies, and a four-dimensional self-efficacy scale for behavior management in the classroom. Results indicate that intrapersonal emotional competencies, as compared to interpersonal competencies, play a major role regarding self-efficacy beliefs. In particular, multiple regression analyses revealed that higher identification and understanding of personal emotions were associated with better perceived self-efficacy on two aspects of SEBiBM. In addition, using other’s emotions predicted proactive involvement of the pupil’s parent or caregiver. Results are discussed in terms of their contribution to research in educational sciences and in teacher education, particularly with respect to teachers’ sustainability in the profession.

## Theoretical framework

Emotional competencies refer to a combination of knowledge, skills, and dispositions related to affect (in the broadest sense of the term, e.g., encompassing emotions, feelings, mood) that enable individuals to effectively and functionally identify, express, understand, use, and regulate one’s own emotions and the emotions of others (Brasseur et al., 2013; Mikolajczak et al., 2020). Emotional competencies have been studied within the emotional intelligence domain, where two main approaches have been introduced: the ability approach, which conceptualizes emotional intelligence as an ability/form of intelligence and measures the construct with tests based on objective performance (Mayer and Salovey, 1997); and the trait approach, which considers emotional intelligence as a personality trait and measures it with self-reports (Petrides and Furnham, 2001). The current contribution was carried out according to the latter approach.

Generally, better emotional competencies contribute to better mental and physical health (Martins et al., 2010), more constructive and satisfying social relationships (Lopes et al., 2003), and greater career success, among other things (e.g., Mikolajczak et al., 2020). In the teaching context, research is increasingly interested in teachers’ social–emotional competencies, particularly in terms of their contribution to learning and to individual and social well-being (Mac Cann et al., 2020; Mikolajczak et al., 2020). Indeed, teachers and pupils experience a wide range of emotions that they have to use or manage during lessons, both in relation to the behaviors of others and in themselves (e.g., frustration, anxiety, but also interest or pride; Hargreaves, 2000; Genoud et al., 2020). Specifically, it can be very challenging for teachers to manage situations in which their own emotions and their pupils’ emotions are obstacles to teaching and learning: fear of coming to school or failing an exam; anger or sadness after a lesson or recess; excessive joy in a play activity, as well as frustration toward pupils’ behavior in class.

Two main negative characteristics of the teaching profession, namely high levels of stress and burnout, have led researchers to explore the role emotional competencies might play in improving teachers’ psychological health and wellbeing (Vesely-Maillefer and Saklofske, 2018). Research shows that emotional competencies may significantly improve teachers’ well-being at work (e.g., Mérida-Lopez and Extremera, 2017), and that training emotional competencies improved teachers’ self-perceived emotional intelligence and interpersonal relationships, lowering the stress level in comparison to the control group (Genoud and Reicherts, 2009; Pérez-Escoda et al., 2012).

Classroom management – also a critical issue in effective teaching and learning – involves several aspects which can be related to teachers’ emotional competencies (Jennings and Greenberg, 2009; Garner, 2010). Evertson and Weinstein (2011, p. 5) characterized classroom management through the five following facets: (1) developing caring and supportive relationships with and among pupils, (2) organizing and implementing instruction in ways that optimize pupils’ access to learning, (3) using group management methods that encourage pupils’ engagement in academic tasks, (4) promoting the development of pupils’ social skills and self-regulation, and (5) using appropriate interventions to assist pupils with behavior problem. The latter refers to the ability to manage behaviors in the classroom; and the subjective perceptions in one’s own SEBiBM – often cited as one of the most challenging aspects of teachers’ education and preservice teachers’ job – constitutes an important risk for burnout, stress, and job (dis)satisfaction in the profession (Evertson and Weinstein, 2011; Dicke et al., 2015). Because SEBiBM is the aspect of classroom management in which teachers encounter the most difficulties (Gaudreau et al., 2018), this study will focus specifically on this multifaceted construct.

In this vein, Dessibourg (2018) validated a four-dimensional instrument to evaluate teacher SEBiBM: (1) proactive management, (2) reactive management, (3) proactive involvement of the pupil’s parent or caregiver, and (4) reactive involvement of people outside the classroom. Proactive management refers to the perceived capacity to establish classroom procedures and routines to prevent occurrence of disruptive behavior (e.g., through the development of classroom rules or clear instructions on how to behave). Reactive management is the perceived teacher’s immediate reaction to pupils’ disruptive or positive behavior (e.g., sanctions or reinforcements). Whereas the first two dimensions refer to perceived management by the teacher alone, the last two assess perceived management in collaboration with adults outside the classroom. The former – named proactive parental involvement – consists in including parents (e.g., in writing, by a telephone call or meeting with the parents) to inform them of how the behavior management system in the classroom functions, thus including them by regularly informing them of their child’s situation in relation to this aspect and, when necessary, by making decisions jointly with them. The latter – named reactive involvement of people outside the classroom – implies involving additional professionals (e.g., the principal, the mediators, the school psychologist) as a resource for the teacher and also for the parents when a situation is difficult to manage.

Different studies investigated the relationships between SEBiBM and emotional competencies. Using one dimensions of the Emmer and Hickman (1991) instrument regarding perceived discipline management in the classroom, Valente et al. (2019) showed – using a structural equation model on a sample of 559 elementary and secondary school teachers – a weak negative relation with emotional perception and a weak positive relation with emotional regulation and perceived classroom discipline management, whereas no relation was found with emotional expression. With the same type of analyses on another sample of 382 teachers (7th to 12th grades), Valente and Lourenço (2020) found that participants with higher emotional intelligence (EI) use more constructive strategies (compromise and collaboration) and fewer inappropriate strategies (domination, avoidance, or obliging). As a result, individuals with high EI tend to be more creative, flexible, and more able to cope with their own emotions, with greater problem-solving and conflict negotiation skills. In a linear regression model, Moafian and Ghanizadeh (2009) showed that emotional self-awareness (i.e., the ability to be aware of, recognize and understand one’s emotions) and interpersonal relationship (i.e., to establish and maintain mutually satisfying relationships and efficiency in problem-solving) predicted a global score of self-efficacy in the teachers of an Iranian private institute – negatively for the first, positively for the two others. Finally, in a longitudinal design, Burić and Mornar (2022) found that positive affectivity (PA) measured by the PANAS (Watson et al., 1988) positively predicted self-efficacy (measured by the Teacher Self-efficacy Scale; Schwarzer et al., 1999) and deep acting (i.e., making efforts to feel emotions needed to display to others), but negatively predicted surface acting (i.e., hiding emotions and faking emotions); while negative affectivity (NA) negatively predicted self-efficacy but positively predicted surface acting. In synthesis, across time, PA was consistently important in predicting self-efficacy while NA was consistently important in predicting surface acting.

Overall, these results support the idea that emotional competencies and authentic expression of emotions allow the teacher to be attuned to the classroom climate and therefore demonstrate better classroom climate management, which would result in stronger perceptions of SEBiBM. Hence, greater awareness would lead to better behavior management by way of greater self-efficacy beliefs.

It is interesting to note that the limited research on the relationship between emotional competencies and SEBiBM has not really taken into account their multidimensional natures, particularly with respect to both intrapersonal and interpersonal emotional competencies. Indeed, most measures have blurred a fundamental distinction between emotional competencies that pertain to the individual (i.e., intrapersonal competencies such as: identification of emotions in oneself) and emotion competencies that apply to another individual (i.e., interpersonal competencies such as: identification of emotions in others). Individuals may hold beliefs about emotional behavior that do not equally refer to others as to themselves. For example, one may know that reappraisal is an emotion regulation strategy that functions well for most individuals and in different contexts, and yet the same person may also know that she or he is not capable of using reappraisal when trying to regulate negative feelings. This is also supported by studies showing that thinking about someone else’s perspective or taking a self-perspective activated partially different neural mechanisms and brain regions (Davis et al., 2004; Vogeley et al., 2004). Hence, the competencies that refer to the intrapersonal or interpersonal sphere may be only loosely related with each other and predict different outcomes. For example, Di Fabio and Palazzeschi (2008) found that a SEBiBM score was positively correlated with the intrapersonal and adaptability scores of emotional competencies, but not related to the interpersonal and stress management scores of emotional competencies.

The purpose of this study was to provide further evidence regarding the association between self-perceived emotional competencies and SEBiBM by using multifaceted scales that distinguish intra-from interpersonal emotional competencies (Brasseur et al., 2013) and four behavior management skills (Dessibourg, 2018) among elementary school teachers.

## Materials and methods

### Participants

The sample comprised 121 teachers from three different regions of the French-speaking part of Switzerland teaching in grade levels from first through fourth grade (117 women and 4 men; *M*_age_ = 41.0, *SD* = 11.2; *M*_years of experience_ = 18.7, *SD* = 12.1). Inclusion criteria were to be native French speaking and having currently a class to teach to. The career paths followed by the participants were tertiary education (University of Teacher Education; *n* = 52) or former degrees called *Écoles Normales* (*n* = 69).

Participants responded voluntarily to a few socio-demographic questions (age, gender, years of experience, educational background and grade level) and then completed the two following instruments: the French version of the Profile of Emotional Competence (PEC; Brasseur et al., 2013), and the French version of the Teacher Self-Efficacy Beliefs in Behavior Management (T-SEBiBM; Dessibourg, 2018).

### Measures

The PEC gives the following 13 indices: a total score of emotional competencies (50 items assessed on a 5-point Likert scale ranging from “strongly disagree” to “strongly agree”), a global score of intrapersonal emotional competencies (25 items) and another score of interpersonal emotional competencies (25 items), and the scores of the emotional sub-competencies, five for each intra and interpersonal competencies (each composed of five items): identification, expression, comprehension, regulation, and utilization of emotions. For all these scores, higher values correspond to better emotional competencies.

In the present study, the subscales’s reliability was good (α from 0.61 to 0.74) except for the subscale “listening to others’ emotions” (α = 0.55); the reliability of the global factors was very good (α from 0.82 to 0.88).

The T-SEBiBM is a questionnaire with 16 items assessed on an 8-point Likert scale (ranging from “I am not at all capable” to “I am completely capable”) and provide four scores: (1) proactive management, (2) reactive management, (3) proactive involvement of the pupil’s parent or caregiver, and (4) reactive involvement of people outside the classroom. For each of these four scores, higher values correspond to higher perceived SEBiBM.

In the present study, the subscales reliability was very good (α from 0.84 to 0.86) except for the subscale “proactive involvement of the pupil’s parent or caregiver” (α = 0.49).

### Data analyses

Statistical analyses were performed with R (R Core Team, 2022). Skewness and kurtosis suggested each variable respected enough normality (all absolute values <1.22). We calculated Pearson’s correlations and four multiple regression analyses with the 10 emotional competences and number of years of experience in teaching as simultaneous predictors of each facet of T-SEBiBM. In the multiple regression analyses, variance inflation factor scores (all <2.31) suggested no strong concern for multicollinearity.

## Results

Means, standard deviations, and correlations with confidence intervals among all variables are available in Supplementary materials; we present here the correlations of interest (Table 1). Facets of the T-SEBiBM showed higher (positive) correlations with intrapersonal emotional competencies than interpersonal competencies, except for using emotions and regulating emotions. Identification, understanding and regulation of one’s emotions generally showed the highest correlations with perceived SEBiBM management, whereas expression of others’ emotions was the only dimension of the PEC unrelated to any aspects of the T-SEBiBM.

**Table 1:** Results of Pearson’s correlations between the 12 scores of emotional competencies and the four dimensions of perceived self-efficacy in classroom management.

Emotional competencies		Proactive management	Reactive management	Proactive involvement	Reactive involvement
Intrapersonal	Identification	0.36**	0.39**	0.35**	0.29**
	Understanding	0.33**	0.36**	0.12	0.23*
	Use	0.19*	0.21*	0.17	0.19*
	Expression	0.25**	0.13	0.10	0.16
	Regulation	0.31**	0.13	0.20*	0.19*
Interpersonal	Identification	0.14	0.18*	0.10	0.24**
	Understanding	0.12	0.22*	0.06	0.13
	Use	0.14	0.07	0.25**	0.09
	Expression	0.04	0.01	−0.08	0.06
	Regulation	0.22*	0.10	0.16	0.19*
Global score	Intrapersonal	0.43**	0.35**	0.28**	0.31**
	Interpersonal	0.19*	0.17	0.15	0.21*

*Indicates *p* < 0.05, **Indicates *p* < 0.01.

For all the four outcomes (i.e., the four dimensions of the T-SEBiBM), results indicated significant predictors among emotional competencies, with percentages of explained variance ranging from 17 to 25% (*p*s < 0.01, Table 2). Understanding of emotions in oneself (intrapersonal competence) emerged as a significant positive predictor of two scores of the T-SEBiBM: Proactive management and reactive management. Identification of one’s emotions (intrapersonal competences) also positively predicted two dimensions of the T-SEBiBM: Reactive management and proactive involvement of caregivers. This latter facet of SEBiBM was also positively predicted by utilizing others’ emotions (interpersonal competence). None of emotional competencies predicted significantly reactive involvement (but trends could be noted).

**Table 2:** Regression results using the four dimensions of perceived self-efficacy in behavior management as outcomes, with number of year of experience and the 10 dimensions of emotional competencies as independent variables.

Predictor	*b*	*b* 95% CI (LL, UL)	Beta	Beta 95% CI (LL, UL)	*sr* ^2^	*sr*^2^ 95% CI (LL, UL)	Fit
*Regression results using proactive management as the outcome*							
(Intercept)	11.46*	(0.84, 22.08)					
Experience	0.04	(−0.05, 0.13)	0.08	(−0.10, 0.26)	0.01	(−0.02, 0.03)	
Identification (intrapersonal)	0.34	(−0.13, 0.81)	0.18	(−0.07, 0.42)	0.01	(−0.02, 0.05)	
Understanding (intrapersonal)	0.41*	(0.02, 0.79)	0.22	(0.01, 0.43)	0.03	(−0.02, 0.08)	
Expression (intrapersonal)	0.12	(−0.27, 0.50)	0.07	(−0.16, 0.30)	0.00	(−0.01, 0.02)	
Regulation (intrapersonal)	0.27	(−0.07, 0.62)	0.17	(−0.04, 0.38)	0.02	(−0.02, 0.06)	
Use (intrapersonal)	0.24	(−0.07, 0.56)	0.14	(−0.04, 0.32)	0.02	(−0.02, 0.06)	
Identification (intrapersonal)	−0.01	(−0.56, 0.54)	−0.00	(−0.26, 0.25)	0.00	(−0.00, 0.00)	
Understanding (interpersonal)	−0.3	(−0.74, 0.15)	−0.15	(−0.38, 0.07)	0.01	(−0.02, 0.05)	
Expression (interpersonal)	−0.13	(−0.57, 0.31)	−0.06	(−0.28, 0.15)	0.00	(−0.01, 0.02)	
Regulation (interpersonal)	0.03	(−0.49, 0.56)	0.02	(−0.23, 0.26)	0.00	(−0.00, 0.00)	
Use (interpersonal)	0.24	(−0.10, 0.58)	0.14	(−0.06, 0.33)	0.01	(−0.02, 0.05)	
							*R*^2^ = 0.245**
							95% CI (0.05, 0.31)
*Regression results using reactive management as the outcome*							
(Intercept)	11.70*	(2.27, 21.13)					
Experience	0.02	(−0.06, 0.09)	0.04	(−0.14, 0.23)	0.00	(−0.01, 0.01)	
Identification (intrapersonal)	0.42*	(0.00, 0.84)	0.25	(0.00, 0.50)	0.03	(−0.02, 0.08)	
Understanding (intrapersonal)	0.41*	(0.07, 0.76)	0.26	(0.04, 0.47)	0.04	(−0.02, 0.10)	
Expression (intrapersonal)	−0.09	(−0.43, 0.25)	−0.06	(−0.30, 0.17)	0.00	(−0.01, 0.02)	
Regulation (intrapersonal)	0.01	(−0.30, 0.31)	0.01	(−0.21, 0.22)	0.00	(−0.00, 0.00)	
Use (intrapersonal)	0.19	(−0.09, 0.47)	0.13	(−0.06, 0.31)	0.01	(−0.02, 0.05)	
Identification (intrapersonal)	−0.02	(−0.51, 0.47)	−0.01	(−0.27, 0.25)	0.00	(−0.00, 0.00)	
Understanding (interpersonal)	0.03	(−0.36, 0.42)	0.02	(−0.22, 0.25)	0.00	(−0.00, 0.00)	
Expression (interpersonal)	−0.04	(−0.43, 0.35)	−0.02	(−0.24, 0.19)	0.00	(−0.01, 0.01)	
Regulation (interpersonal)	−0.13	(−0.60, 0.33)	−0.07	(−0.32, 0.18)	0.00	(−0.01, 0.02)	
Use (interpersonal)	0.15	(−0.15, 0.46)	0.10	(−0.10, 0.30)	0.01	(−0.02, 0.03)	
							*R*^2^ = 0.214**
							95% CI (0.03, 0.27)
*Regression results using proactive involvement as the outcome*							
(Intercept)	3.8	(−0.85, 8.46)					
Experience	0.02	(−0.01, 0.06)	0.12	(−0.06, 0.30)	0.01	(−0.02, 0.05)	
Identification (intrapersonal)	0.30**	(0.10, 0.51)	0.36	(0.12, 0.61)	0.06	(−0.01, 0.13)	
Understanding (intrapersonal)	−0.02	(−0.19, 0.15)	−0.02	(−0.23, 0.19)	0.00	(−0.01, 0.01)	
Expression (intrapersonal)	−0.05	(−0.21, 0.12)	−0.06	(−0.29, 0.17)	0.00	(−0.01, 0.02)	
Regulation (intrapersonal)	0.1	(−0.05, 0.25)	0.14	(−0.07, 0.35)	0.01	(−0.02, 0.04)	
Use (intrapersonal)	0.09	(−0.05, 0.23)	0.11	(−0.07, 0.30)	0.01	(−0.02, 0.04)	
Identification (intrapersonal)	0.05	(−0.19, 0.29)	0.05	(−0.20, 0.30)	0.00	(−0.01, 0.01)	
Understanding (interpersonal)	−0.16	(−0.35, 0.03)	−0.19	(−0.42, 0.04)	0.02	(−0.02, 0.06)	
Expression (interpersonal)	−0.11	(−0.30, 0.08)	−0.12	(−0.33, 0.09)	0.01	(−0.02, 0.04)	
Regulation (interpersonal)	0.02	(−0.21, 0.25)	0.02	(−0.22, 0.26)	0.00	(−0.00, 0.00)	
Use (interpersonal)	0.15*	(0.00, 0.30)	0.20	(0.00, 0.39)	0.03	(−0.02, 0.08)	
							*R*^2^ = 0.243**
							95% CI (0.05, 0.30)
*Regression results using reactive involvement as the outcome*							
(Intercept)	3.64	(−3.91, 11.18)					
Experience	0.06	(−0.01, 0.12)	0.17	(−0.02, 0.36)	0.02	(−0.03, 0.07)	
Identification (intrapersonal)	0.14	(−0.19, 0.47)	0.11	(−0.15, 0.37)	0.01	(−0.02, 0.03)	
Understanding (intrapersonal)	0.12	(−0.15, 0.39)	0.10	(−0.12, 0.31)	0.01	(−0.02, 0.03)	
Expression (intrapersonal)	−0.06	(−0.33, 0.21)	−0.05	(−0.30, 0.19)	0.00	(−0.01, 0.01)	
Regulation (intrapersonal)	0.12	(−0.12, 0.37)	0.11	(−0.11, 0.33)	0.01	(−0.02, 0.04)	
Use (intrapersonal)	0.16	(−0.07, 0.38)	0.13	(−0.06, 0.32)	0.01	(−0.02, 0.05)	
Identification (intrapersonal)	0.38	(−0.01, 0.77)	0.26	(−0.01, 0.52)	0.03	(−0.03, 0.08)	
Understanding (interpersonal)	−0.19	(−0.50, 0.13)	−0.14	(−0.38, 0.10)	0.01	(−0.02, 0.04)	
Expression (interpersonal)	−0.1	(−0.41, 0.21)	−0.07	(−0.30, 0.15)	0.00	(−0.02, 0.02)	
Regulation (interpersonal)	0.07	(−0.30, 0.44)	0.05	(−0.21, 0.31)	0.00	(−0.01, 0.01)	
Use (interpersonal)	0.02	(−0.22, 0.26)	0.02	(−0.19, 0.22)	0.00	(−0.00, 0.00)	
							*R*^2^ = 0.167*
							95% CI (0.00, 0.22)

A significant *b*-weight indicates the beta-weight and semi-partial correlation are also significant. *b* represents unstandardized regression weights. Beta indicates the standardized regression weights. *sr*^2^ represents the semi-partial correlation squared. *LL* and *UL* indicate the lower and upper limits of a confidence interval, respectively. *Indicates *p* < 0.05, **indicates *p* < 0.01.

## Discussion

The goal of the current study was to understand which facets of emotional competencies may foster specific aspects of perceived classroom management efficacy, focusing on behavior management. Results show that intrapersonal, more than interpersonal emotional competencies play a major role in teachers’ self-perceptions regarding behavior management. Consequently, SEBiBM may primarily relate to self-management capacities or self-confidence regarding one’s own emotions. This is consistent with Di Fabio and Palazzeschi (2008) results showing that SEBiBM was positively correlated with the intrapersonal, but not with the interpersonal dimension of emotional competencies. Our research extends these results by identifying specific intrapersonal competencies that could help teachers in managing pupils’ behavior within the classroom. The lack of correlations between interpersonal aspects and self-efficacy beliefs could also be partially explained by the fact that teacher self-efficacy seems to stabilize early on, meaning it should not respond rapidly to external factors (Lazarides et al., 2020).

Starting from perceived self-efficacy in proactive management, the dimension of the T-SEBIBM that refers to setting up strategies to prevent disruptive behavior in class, understanding emotion (intrapersonal emotional competence) predicted this type of management strategy. This result suggests that being able to locate causes and consequences of own emotion help to foresight and adopt appropriate strategies for behavior management. Teachers may rely on understanding the causes of their emotion and associated need to foresee potential affective states (and in particular stress) that pupil misbehavior is likely to generate. Teachers being able to locate one’s causes and consequences of emotions in an adequate way rather than just the trigger of affects may also be more consistent between their values (e.g., each student can show progress) and the actions implemented, showing a caring approach (Shankland et al., 2018). Consequently, good competencies in understanding emotions may allow a more serene consideration of class dynamics and reduce teachers’ stress and exhaustion, similarly to what has been reported for a better emotional regulation (Vesely-Maillefer and Saklofske, 2018).

Regarding the reactive management strategy, the dimension of the T-SEBIBM that refers to directly reacting to pupils’ behavior in class, we found that identifying one’s own emotions and understanding them were both significant predictors. On one hand, it seems that being aware of how one is feeling may help to decide and control how to respond to both positive (e.g., with reinforce) and negative behavior (with punishment) better. On the other hand, the better teachers understand the causes and consequences of their emotions, the more efficient they are to react to pupils’ behavior whether it is disruptive or positive. Indeed, when teachers have to deal with unwanted behavior in the classroom and encourage positive behavior, they are undoubtedly confronted to certain emotions (e.g., the anxiety of perceiving the classroom climate deteriorate, of losing authority during the intervention; and also the joy or the interest in seeing a positive climate); the ability to identify the affective state they are experiencing and the understanding of their causes and consequences (i.e., interrupting their teaching to intervene in the face of a momentary difficulty or to emphasize good behavior) seems to be a useful tool for maintaining a feeling of effectiveness in this type of intervention. Possibly, teachers’ competencies with their own emotions may translate to a better understanding or deducting more accurately the causes and consequences of pupils’ emotions in the classroom; this might help to infer what caused a pupil’s reaction and interfere more effectively to address or prevent any consequences (or amplify them if they are positive).

As suggested in previous studies (Chang, 2009; Gay and Genoud, 2020), understanding the causes and consequences of emotions may facilitate decision making in the classroom and help teachers think in a more constructive way. For example, instead of feeling angry or anxious when a pupil does not follow classroom rules or refuses to work, teachers who understand their own emotions better may be more apt at re-evaluating the situation by putting it into perspective or by promoting positive attitude toward pupils (interpret pupils’ behaviors as personal problems rather than a personal attitude toward the teacher). This could facilitate reacting to disruptive behaviors as well as preventing them.

In a similar way, identifying emotions may help teachers manage their classroom and involve pupil’s parents in a more proactive way, such as through recognizing mild anger early and managing it adequately before it potentially escalates into fury. If identifying unpleasant emotions allows one to become aware of something that needs to be changed or of the need to ask for a helping hand, identifying pleasant emotions can promote enthusiasm, decision making and broaden possible perspectives (Fredrickson and Branigan, 2005).

When it comes to the dimension of proactive involvement of caregiver – which also showed a marked percentage of explained variance – it is the only variable for which one of the interpersonal factor score was significant. Indeed, in addition to identifying one’s own emotions, also using others’ emotions to reflect, act and make decisions better emerged as a requisite for teachers to feel that they are able to establish effective collaborations with parents. Including adults outside the classroom by using others’ emotions may be important to help teachers in involving parents and knowing what to do when it comes to motivating and collaborating with them.

Finally, the last dimension – reactive involvement of external people, such as the school principal or a psychologist – had a lower explained percentage of variance than the other three dimensions. This facet of T-SEBiBM was not predicted by any variables but the identification of emotions in oneself (intrapersonal emotional competencies) approached significance. Although one might have expected some interpersonal dimensions to be significant, it might be that the teacher loses some control – and therefore a significant part of his or her sense of self-efficacy – in situations where other professionals have to intervene. Indeed, in such situations, the school principal or the mediator are certainly the people who take charge of the problem reported by the teacher (who realizes there is a problem to solve by identifying his or her own emotional reactions and calls on them because the situation is beyond his or her power). In this case, teachers’ own emotional competencies may have less impact on self-efficacy beliefs, given that classroom management in this context is partially beyond their control.

Overall, even though all intrapersonal emotional competencies were correlated to proactive management, understanding and identification revealed to be the stronger importance to SEBiBM. In particular, being able to identify and understand one’s own affective state were the most important predictors of a teacher’s self-efficacy regarding behavior management, including three out of four subdimensions. Indeed, identifying and understanding of one’s own emotions are key emotional competencies in various models because they are considered prerequisite for the ability to use and regulate emotions (Mikolajczak et al., 2020). Finally, expression of others’ emotions – to allow others to express their emotions – was the only emotional competence uncorrelated to any of the four of aspects of perceived self-efficacy in behavior management.

### Theoretical and practical implications

These results have important implications from both a theoretical and a practical perspective. From a theoretical point of view, this study distinguishes the contribution of intrapersonal and interpersonal emotional competencies, two dimensions that are often blurred in emotional competencies research despite being quite distinct from each other (Mikolajczak et al., 2020), as our results also showed. In addition, this is the first study, to our knowledge, connecting specific emotional competencies to different dimensions of behavior management. Results highlight the prominent role of identifying and understanding one’s own emotions as fundamental skills teachers need to have in order to feel confident in managing the class effectively. Other emotional competencies show specific associations to specific self-efficacy beliefs related to different emotion management strategies, highlighting the usefulness of using a multifaceted approach in conceptualizing and measuring emotional competencies.

From a practical perspective, these findings reinforce the interest around the development of pre-service and in-service training to develop teachers’ emotional competencies. This training should be based on an understanding of the specific affective processes underlying teacher self-efficacy beliefs in behavior management. The present findings suggest that different emotional competencies play an important role in helping teachers feel better within their class as well as in their professional development and related self-confidence, to prevent indiscipline, respond to pupil behavior in an appropriate manner, and engage professionals and caregivers beyond the classroom. Thus, training that encourages the development of these emotional competencies may be effective for perceived self-efficacy classroom management, which constitutes a critical issue for effective teaching and learning (Muijs and Reynolds, 2002).

### Limitations and future directions

The main limitations of the current work are the reliance on self-report, the small sample size, predominantly constituted of female teachers, and the correlational design which precludes any inference of causality. We assume that emotional competencies, which are individual characteristics that have an inborn component, although trainable, would precede the development of SEBiBM. However, we cannot be certain of the direction of the association given the cross-sectional design and we could also imagine that having stronger SEBiBM influences one’s own perceptions about emotional competence.

Future research should use longitudinal designs to address the issue of causality. As an extension of the present results, other facets of classroom management (e.g., developing caring and supportive relationships with and among pupils, promoting the development of pupils’ social skills and self-regulation) could enlighten the role of emotional competencies in teaching and learning. It would also be interesting in future studies to use more objective measures of teachers’ effectiveness (e.g., collecting pupils’ evaluations, direct observations of certain behaviors) to see possible discrepancies with self-reported competencies. Despite objective indicators of teachers’ effectiveness, subjective perceptions in one’s own competencies and abilities to manage the class, or self-efficacy beliefs, remain a key aspect to investigate in the teaching profession because they are related to important outcomes, including students’ achievement (Collie et al., 2012) and teachers’ burnout (Gholami, 2015).

Results can thus be considered as a promising preliminary study providing a pathway for future research, which should include attention to evaluating these relationships prospectively, through mixed assessments (e.g., subjective scales, more objective tests of emotional competencies), and considering multiple perspectives (e.g., teachers, principals, and pupils) in order to be able to bring about a higher level of evidence. Future work could also compare the effects of different programs on developing emotional competencies in teachers – as a lever to initiate different changes in professional development – to determine the extent to which some trainings are more appropriate than others, depending on classes, pupils or teachers’ characteristics. Our results show that a training based on developing emotional competencies in oneself – as compared to competencies that help understand, identify and manage emotions in others – could be promising regarding teachers’ ways of managing pupils and their behavior within the classroom. Teachers are often confronted to strong emotional reactions (stress, frustration, anger) and learning to recognize such emotions in the first place could be a fundamental step toward allowing the development of more complex emotion regulation strategies.

## Conclusion

Which are the most important competencies for teachers to feel more effective in specific aspects of their behavior management? The present study investigated different general (i.e., intra-and interpersonal) and specific (e.g., identifying, understanding) emotional competencies of teachers as associated with efficacy beliefs regarding distinct behavior management dimensions. It showed that intrapersonal emotional competencies, specifically identification and understanding of emotions in oneself, play a fundamental role in fostering self-efficacy perceptions regarding different class management strategies.

Beyond offering a contribution to the research area of educational sciences, this study highlights the relevance of training emotional competencies for both in-service and pre-service teachers. Future studies could refine these findings and provide a deeper understanding of the importance of emotional competencies for classroom management, possibly by enlightening the role of potential mediating mechanisms, such as teachers’ self-efficacy beliefs.

## Data availability statement

The raw data supporting the conclusions of this article will be made available by the authors, without undue reservation.

## Ethics statement

Ethical review and approval was not required for the study on human participants in accordance with the local legislation and institutional requirements. Written informed consent for participation was not required for this study in accordance with the national legislation and the institutional requirements.

## Author contributions

PGa wrote the first draft of the manuscript and the final version. MF and PGe revised and wrote sections of the manuscript. PGa and PGe did the statistical analyses. MB revised English and several paragraphs related to classroom management. GK, J-MG, and MC contributed to general conceptualization of the manuscript. All authors contributed to the article and approved the submitted version.

## Funding

Open access funding provided by Haute École Pédagogique Du Canton De Vaud (HEP Vaud).

## Conflict of interest

The authors declare that the research was conducted in the absence of any commercial or financial relationships that could be construed as a potential conflict of interest.

## Publisher’s note

All claims expressed in this article are solely those of the authors and do not necessarily represent those of their affiliated organizations, or those of the publisher, the editors and the reviewers. Any product that may be evaluated in this article, or claim that may be made by its manufacturer, is not guaranteed or endorsed by the publisher.

## References

[ref1] Brasseur S. Grégoire J. Bourdu R. Mikolajczak M. (2013). The profile of emotional competence (PEC): development and validation of a self-reported measure that fits dimensions of emotional competence theory. PLoS One 8:e62635. doi: 10.1371/journal.pone.0062635 23671616 PMC3646043

[ref2] Burić I. Mornar M. (2022). Teacher dispositional affectivity, emotional labor, and self-efficacy: a longitudinal analysis. Curr. Psychol. doi: 10.1007/s12144-022-03029-7

[ref3] Chang M.-L. (2009). An appraisal perspective of teacher burnout: examining the emotional work of teachers. Educ. Psychol. Rev. 21, 193–218. doi: 10.1007/s10648-009-9106-y

[ref4] Collie R. J. Shapka J. D. Perry N. E. (2012). School climate and social–emotional learning: predicting teacher stress, job satisfaction, and teaching efficacy. J. Educ. Psychol. 104, 1189–1204. doi: 10.1037/a0029356

[ref5] Davis M. H. Soderlund T. Cole J. Gadol E. Kute M. Myers M.. (2004). Cognitions associated with attempts to empathize: how do we imagine the perspective of another? Personal. Soc. Psychol. Bull. 30, 1625–1635. doi: 10.1177/0146167204271183 15536244

[ref6] Dessibourg M. S. (2018). Sentiment d’efficacité personnelle en gestion des comportements. Elaboration et validation d’une échelle de mesure. Revue Suisse des Sciences de l'Education 40, 697–724. doi: 10.24452/sjer.40.3.5124

[ref7] Di Fabio A. Palazzeschi L. (2008). Emotional intelligence and self-efficacy in a sample of Italian high school teachers. Soc. Behav. Personal. Int. J. 36, 315–326. doi: 10.2224/sbp.2008.36.3.315

[ref8] Dicke T. Elling J. Schmeck A. Leutner D. (2015). Reducing reality shock: the effects of classroom management skills training on beginning teachers. Teach. Teach. Educ. 48, 1–12. doi: 10.1016/j.tate.2015.01.013

[ref9] Emmer E. T. Hickman J. (1991). Teacher efficacy in classroom management and discipline. Educ. Psychol. Meas. 51, 755–765. doi: 10.1177/0013164491513027

[ref10] Evertson C. M. Weinstein C. S. (2011). Handbook of Classroom Management: Research, Practice, and Contemporary Issues Routledge.

[ref11] Fredrickson B. L. Branigan C. (2005). Positive emotions broaden the scope of attention and thought-action repertoires. Cognit. Emot. 19, 313–332. doi: 10.1080/02699930441000238 21852891 PMC3156609

[ref12] Garner P. W. (2010). Emotional competence and its influences on teaching and learning. Educ. Psychol. Rev. 22, 297–321. doi: 10.1007/s10648-010-9129-4

[ref13] Gaudreau N. Verret C. Massé L. Nadeau M.-F. Picher M. J. (2018). La scolarisation des élèves présentant des difficultés comportementales: analyse écologique des conditions relatives à leur intégration au secondaire. Revue Canadienne de L’éducation 41, 555–583.

[ref14] Gay P. Genoud P. A. (2020). Quelles compétences émotionnelles protègent des différentes dimensions du burnout chez les enseignants du primaire? Recherches en Éducation 41, 74–91. doi: 10.4000/ree.572

[ref15] Genoud P. A. Kappeler G. Gay P. (2020). Faut-il former les enseignants afin qu’ils cherchent à diminuer les émotions négatives de leurs élèves ou qu’ils leur apprennent à renforcer leurs émotions positives? Recherches en Éducation 41, 31–45. doi: 10.4000/ree.519

[ref16] Genoud P. A. Reicherts M. (2009). “Emotional openness as a protective factor against burnout,” in Psychology of Burnout: Predictors and Coping Mechanisms. ed. Schwartzhoffer R. V. (Nova Science Publishers), 167–181.

[ref17] Gholami L. (2015). Teacher self-efficacy and teacher burnout: a study of relations. Int. Lett. Soc. Humanist. Sci. 60, 83–86. doi: 10.18052/www.scipress.com/ILSHS.60.83

[ref18] Hargreaves A. (2000). Mixed emotions: teachers’ perceptions of their interactions with students. Teach. Teach. Educ. 16, 811–826. doi: 10.1016/S0742-051X(00)00028-7

[ref19] Jennings P. A. Greenberg M. T. (2009). The prosocial classroom: teacher social and emotional competence in relation to student and classroom outcomes. Rev. Educ. Res. 79, 491–525. doi: 10.3102/0034654308325693

[ref20] Lazarides R. Watt H. M. Richardson P. W. (2020). Teachers’ classroom management self-efficacy, perceived classroom management and teaching contexts from beginning until mid-career. Learn. Instr. 69:101346. doi: 10.1016/j.learninstruc.2020.101346

[ref21] Lopes P. N. Salovey P. Straus R. (2003). Emotional intelligence, personality and the perceived quality of social relationships. Personal. Individ. Differ. 35, 641–659. doi: 10.1016/S0191-8869(02)00242-8

[ref22] Mac Cann C. Jiang Y. Brown L. E. R. Double K. S. Bucich M. Minbashian A. (2020). Emotional intelligence predicts academic performance: a meta-analysis. Psychol. Bull. 146, 150–186. doi: 10.1037/bul0000219 31829667

[ref23] Martins A. Ramalho N. Morin E. (2010). A comprehensive meta-analysis of the relationship between emotional intelligence and health. Personal. Individ. Differ. 49, 554–564. doi: 10.1016/j.paid.2010.05.029

[ref24] Mayer J. D. Salovey P. (1997). “What is emotional intelligence?” in Emotional Development and Emotional Intelligence: Educational Implications. eds. Salovey P. Sluyter D. (New York: Basic Books), 3–31.

[ref200] Mérida-López S. Extremera N. (2017). Emotional intelligence and teacher burnout: A systematic review. International Journal of Educational Research, 85, 121–130. doi: 10.1016/j.ijer.2017.07.006

[ref25] Mikolajczak M. Quoidbach J. Kotsou I. Nélis D. (2020). Les compétences émotionnelles. Dunod. doi: 10.3917/dunod.mikol.2020.01

[ref26] Moafian F. Ghanizadeh A. (2009). The relationship between Iranian EFL teachers’ emotional intelligence and their self-efficacy in language institutes. System 37, 708–718. doi: 10.1016/j.system.2009.09.014

[ref27] Muijs D. Reynolds D. (2002). Teachers’ beliefs and behaviors: what really matters? J. Classr. Interact. 37, 3–15.

[ref28] Pérez-Escoda N. Filella G. Alegre A. Bisquerra R. (2012). Developing the emotional competence of teachers and pupils in school contexts. Electron. J. Res. Educ. Psychol. 10, 1183–1207. doi: 10.25115/ejrep.v10i28.1530

[ref29] Petrides K. V. Furnham A. (2001). Trait emotional intelligence: psychometric investigation with reference to established trait taxonomies. Eur. J. Personal. 15, 425–448. doi: 10.1002/per.416

[ref30] R Core Team (2022). R: A Language and Environment for Statistical Computing. R Foundation for Statistical Computing, Vienna, Austria.

[ref31] Schwarzer R. Schmitz G. S. Daytner G. T. (1999). The Teacher Self-Efficacy Scale. Available at: http://userpage.fu-berlin.de/gesund/skalen/Language_Selection/Turkish/Teacher_Self-Efficacy/teacher_self-efficacy.htm (Accessed November 28. 2022).

[ref32] Shankland R. Bressoud N. Tessier D. Gay P. (2018). La bienveillance: Une compétence socio-émotionnelle de l’enseignant au service du bien-être et des apprentissages? Questions Vives. Recherches en Éducation 29, 1–23. doi: 10.4000/questionsvives.3601

[ref33] Valente S. Lourenço A. A. (2020). Conflict in the classroom: how teachers’ emotional intelligence influences conflict management. Front. Educ.:5. doi: 10.3389/feduc.2020.00005

[ref34] Valente S. Monteiro A. P. Lourenço A. A. (2019). The relationship between teachers’ emotional intelligence and classroom discipline management. Psychol. Sch. 56, 741–750. doi: 10.1002/pits.22218

[ref35] Vesely-Maillefer A. K. Saklofske D. H. (2018). Emotional Intelligence and the Next Generation of Teachers. In Emotional Intelligence in Education, eds. Keefer, K., Parker, J., Saklofske, D. (Springer, Cham: The Springer Series on Human Exceptionality). doi: 10.1007/978-3-319-90633-1_14

[ref36] Vogeley K. May M. Ritzl A. Falkai P. Zilles K. Fink G. R. (2004). Neural correlates of first-person perspective as one constituent of human self-consciousness. J. Cogn. Neurosci. 16, 817–827. doi: 10.1162/089892904970799 15200709

[ref37] Watson D. Clark L. A. Tellegen A. (1988). Development and validation of brief measures of positive and negative affect: the PANAS scales. J. Pers. Soc. Psychol. 54, 1063–1070. doi: 10.1037/0022-3514.54.6.1063 3397865

