# Supplementary material for: On the rocky road to efficient behavior management: Can emotional competencies signal the better way?
Source: Front Psychol. 2022 Dec 21;13:1049617. doi: 10.3389/fpsyg.2022.1049617 (PMC9811141; doi:10.3389/fpsyg.2022.1049617)
Supplement: Supplementary file 1 [file Table_1.docx]

Table 3 (supplementary material)

*Means, standard deviations, and correlations with confidence intervals*

| Variable | *M* | *SD* | 1 | 2 | 3 | 4 | 5 | 6 | 7 | 8 | 9 | 10 | 11 | 12 | 13 | 14 | 15 | 16 | 17 | 18 | 19 |
| --- | --- | --- | --- | --- | --- | --- | --- | --- | --- | --- | --- | --- | --- | --- | --- | --- | --- | --- | --- | --- | --- |
|  |  |  |  |  |  |  |  |  |  |  |  |  |  |  |  |  |  |  |  |  |  |
| 1. Age | 40.98 | 11.18 |  |  |  |  |  |  |  |  |  |  |  |  |  |  |  |  |  |  |  |
|  |  |  |  |  |  |  |  |  |  |  |  |  |  |  |  |  |  |  |  |  |  |
| 2. Experience | 18.74 | 12.11 | .98** |  |  |  |  |  |  |  |  |  |  |  |  |  |  |  |  |  |  |
|  |  |  | [.96, .98] |  |  |  |  |  |  |  |  |  |  |  |  |  |  |  |  |  |  |
|  |  |  |  |  |  |  |  |  |  |  |  |  |  |  |  |  |  |  |  |  |  |
| 3. PEC: Global Score | 3.64 | 0.36 | .04 | .02 |  |  |  |  |  |  |  |  |  |  |  |  |  |  |  |  |  |
|  |  |  | [-.14, .22] | [-.16, .19] |  |  |  |  |  |  |  |  |  |  |  |  |  |  |  |  |  |
|  |  |  |  |  |  |  |  |  |  |  |  |  |  |  |  |  |  |  |  |  |  |
| 4. PEC: Intrapersonal Score | 3.74 | 0.44 | .12 | .10 | .89** |  |  |  |  |  |  |  |  |  |  |  |  |  |  |  |  |
|  |  |  | [-.06, .29] | [-.08, .27] | [.85, .92] |  |  |  |  |  |  |  |  |  |  |  |  |  |  |  |  |
|  |  |  |  |  |  |  |  |  |  |  |  |  |  |  |  |  |  |  |  |  |  |
| 5. PEC: Interpersonal Score | 3.54 | 0.39 | -.06 | -.08 | .86** | .54** |  |  |  |  |  |  |  |  |  |  |  |  |  |  |  |
|  |  |  | [-.24, .12] | [-.26, .10] | [.81, .90] | [.40, .66] |  |  |  |  |  |  |  |  |  |  |  |  |  |  |  |
|  |  |  |  |  |  |  |  |  |  |  |  |  |  |  |  |  |  |  |  |  |  |
| 6. PEC: Identification (self) | 3.99 | 0.61 | .14 | .13 | .68** | .76** | .42** |  |  |  |  |  |  |  |  |  |  |  |  |  |  |
|  |  |  | [-.04, .31] | [-.05, .30] | [.57, .77] | [.67, .83] | [.26, .56] |  |  |  |  |  |  |  |  |  |  |  |  |  |  |
|  |  |  |  |  |  |  |  |  |  |  |  |  |  |  |  |  |  |  |  |  |  |
| 7. PEC: Identification (other) | 4.00 | 0.53 | -.17 | -.21* | .69** | .48** | .75** | .47** |  |  |  |  |  |  |  |  |  |  |  |  |  |
|  |  |  | [-.34, .01] | [-.38, -.03] | [.59, .78] | [.33, .61] | [.66, .82] | [.32, .60] |  |  |  |  |  |  |  |  |  |  |  |  |  |
|  |  |  |  |  |  |  |  |  |  |  |  |  |  |  |  |  |  |  |  |  |  |
| 8. PEC: Understanding (self) | 4.07 | 0.62 | .02 | .04 | .51** | .66** | .22* | .53** | .33** |  |  |  |  |  |  |  |  |  |  |  |  |
|  |  |  | [-.16, .20] | [-.14, .21] | [.37, .63] | [.54, .75] | [.04, .38] | [.39, .65] | [.16, .48] |  |  |  |  |  |  |  |  |  |  |  |  |
|  |  |  |  |  |  |  |  |  |  |  |  |  |  |  |  |  |  |  |  |  |  |
| 9. PEC: Understanding (other) | 3.85 | 0.60 | -.07 | -.06 | .70** | .55** | .68** | .50** | .60** | .30** |  |  |  |  |  |  |  |  |  |  |  |
|  |  |  | [-.24, .11] | [-.23, .12] | [.59, .78] | [.42, .67] | [.57, .77] | [.35, .62] | [.47, .70] | [.13, .45] |  |  |  |  |  |  |  |  |  |  |  |
|  |  |  |  |  |  |  |  |  |  |  |  |  |  |  |  |  |  |  |  |  |  |
| 10. PEC: Expression (self) | 3.72 | 0.67 | -.00 | -.03 | .50** | .50** | .36** | .28** | .29** | .10 | .35** |  |  |  |  |  |  |  |  |  |  |
|  |  |  | [-.18, .18] | [-.21, .15] | [.35, .62] | [.35, .62] | [.20, .51] | [.11, .44] | [.12, .44] | [-.07, .28] | [.18, .50] |  |  |  |  |  |  |  |  |  |  |
|  |  |  |  |  |  |  |  |  |  |  |  |  |  |  |  |  |  |  |  |  |  |
| 11. PEC: Expression/Listening (other) | 2.53 | 0.67 | .05 | .02 | .34** | .06 | .56** | .09 | .20* | -.15 | .15 | .12 |  |  |  |  |  |  |  |  |  |
|  |  |  | [-.13, .23] | [-.16, .20] | [.17, .49] | [-.12, .24] | [.43, .67] | [-.09, .27] | [.03, .37] | [-.32, .03] | [-.03, .32] | [-.06, .29] |  |  |  |  |  |  |  |  |  |
|  |  |  |  |  |  |  |  |  |  |  |  |  |  |  |  |  |  |  |  |  |  |
| 12. PEC: Regulation (self) | 3.72 | 0.69 | .02 | -.01 | .71** | .75** | .48** | .49** | .45** | .33** | .43** | .17 | .02 |  |  |  |  |  |  |  |  |
|  |  |  | [-.15, .20] | [-.19, .17] | [.61, .79] | [.65, .82] | [.34, .61] | [.34, .61] | [.30, .58] | [.16, .48] | [.27, .57] | [-.01, .33] | [-.16, .20] |  |  |  |  |  |  |  |  |
|  |  |  |  |  |  |  |  |  |  |  |  |  |  |  |  |  |  |  |  |  |  |
| 13. PEC: Regulation (other) | 4.00 | 0.56 | -.05 | -.01 | .53** | .31** | .64** | .07 | .37** | .13 | .29** | .23** | .05 | .35** |  |  |  |  |  |  |  |
|  |  |  | [-.22, .13] | [-.19, .17] | [.38, .64] | [.14, .46] | [.52, .73] | [-.11, .25] | [.21, .52] | [-.05, .30] | [.11, .44] | [.06, .40] | [-.13, .23] | [.18, .50] |  |  |  |  |  |  |  |
|  |  |  |  |  |  |  |  |  |  |  |  |  |  |  |  |  |  |  |  |  |  |
| 14. PEC: Use (self) | 3.20 | 0.71 | .21* | .20* | .57** | .67** | .31** | .29** | .09 | .28** | .27** | .12 | .11 | .47** | .21* |  |  |  |  |  |  |
|  |  |  | [.03, .38] | [.03, .37] | [.43, .68] | [.56, .76] | [.14, .47] | [.12, .45] | [-.09, .26] | [.11, .44] | [.09, .43] | [-.06, .29] | [-.07, .28] | [.32, .60] | [.04, .38] |  |  |  |  |  |  |
|  |  |  |  |  |  |  |  |  |  |  |  |  |  |  |  |  |  |  |  |  |  |
| 15. PEC: Use (other) | 3.33 | 0.54 | -.01 | -.04 | .71** | .49** | .77** | .32** | .44** | .20* | .30** | .26** | .40** | .44** | .51** | .40** |  |  |  |  |  |
|  |  |  | [-.19, .17] | [-.21, .14] | [.61, .79] | [.34, .62] | [.69, .83] | [.15, .47] | [.29, .57] | [.02, .37] | [.13, .46] | [.08, .42] | [.23, .54] | [.29, .58] | [.37, .63] | [.24, .54] |  |  |  |  |  |
|  |  |  |  |  |  |  |  |  |  |  |  |  |  |  |  |  |  |  |  |  |  |
| 16. TSBiBM: Global Score | 5.66 | 0.94 | .14 | .16 | .36** | .41** | .21* | .40** | .19* | .32** | .17 | .22* | .14 | .20* | .02 | .25** | .19* |  |  |  |  |
|  |  |  | [-.03, .31] | [-.02, .33] | [.20, .51] | [.25, .55] | [.03, .38] | [.24, .54] | [.02, .36] | [.15, .48] | [-.01, .33] | [.04, .38] | [-.04, .31] | [.02, .36] | [-.16, .20] | [.07, .41] | [.02, .36] |  |  |  |  |
|  |  |  |  |  |  |  |  |  |  |  |  |  |  |  |  |  |  |  |  |  |  |
| 17. TSBiBM: Proactive management | 5.55 | 0.96 | .14 | .16 | .36** | .43** | .19* | .36** | .14 | .33** | .12 | .19* | .14 | .25** | .04 | .31** | .22* | .94** |  |  |  |
|  |  |  | [-.04, .31] | [-.02, .33] | [.20, .51] | [.27, .56] | [.02, .36] | [.19, .51] | [-.04, .31] | [.16, .48] | [-.06, .30] | [.01, .36] | [-.04, .31] | [.07, .41] | [-.14, .22] | [.14, .46] | [.04, .38] | [.91, .96] |  |  |  |
|  |  |  |  |  |  |  |  |  |  |  |  |  |  |  |  |  |  |  |  |  |  |
| 18. TSBiBM: Reactive management | 5.98 | 1.01 | .05 | .09 | .30** | .35** | .17 | .39** | .18* | .35** | .22* | .20* | .07 | .13 | .01 | .13 | .10 | .87** | .76** |  |  |
|  |  |  | [-.13, .22] | [-.09, .26] | [.13, .46] | [.19, .50] | [-.01, .34] | [.23, .53] | [.01, .35] | [.19, .50] | [.04, .38] | [.03, .37] | [-.11, .25] | [-.05, .30] | [-.17, .18] | [-.05, .30] | [-.08, .27] | [.83, .91] | [.67, .83] |  |  |
|  |  |  |  |  |  |  |  |  |  |  |  |  |  |  |  |  |  |  |  |  |  |
| 19. TSBiBM: Proactive involvement | 5.12 | 1.27 | .19* | .19* | .25** | .28** | .15 | .35** | .10 | .12 | .06 | .16 | .25** | .10 | -.08 | .20* | .16 | .75** | .66** | .52** |  |
|  |  |  | [.01, .35] | [.02, .36] | [.07, .41] | [.11, .44] | [-.03, .32] | [.19, .50] | [-.08, .27] | [-.06, .30] | [-.12, .24] | [-.01, .33] | [.07, .41] | [-.08, .27] | [-.25, .10] | [.03, .37] | [-.01, .33] | [.66, .82] | [.55, .75] | [.38, .64] |  |
|  |  |  |  |  |  |  |  |  |  |  |  |  |  |  |  |  |  |  |  |  |  |
| 20. TSBiBM: Reactive involvement | 5.69 | 1.30 | .16 | .16 | .30** | .31** | .21* | .29** | .24** | .23* | .13 | .19* | .09 | .15 | .06 | .19* | .19* | .84** | .72** | .61** | .59** |
|  |  |  | [-.02, .33] | [-.02, .33] | [.13, .45] | [.14, .46] | [.03, .37] | [.11, .44] | [.06, .40] | [.05, .39] | [-.05, .30] | [.02, .36] | [-.09, .26] | [-.02, .32] | [-.12, .24] | [.01, .35] | [.01, .36] | [.78, .89] | [.62, .80] | [.49, .71] | [.45, .69] |
|  |  |  |  |  |  |  |  |  |  |  |  |  |  |  |  |  |  |  |  |  |  |

*Note.* *M* and *SD* are used to represent mean and standard deviation, respectively. Values in square brackets indicate the 95% confidence interval for each correlation. The confidence interval is a plausible range of population correlations that could have caused the sample correlation (Cumming, 2014). * indicates *p* < .05. ** indicates *p* < .01.
